# Effects of Sustained Sleep Restriction on Mitogen-Stimulated Cytokines, Chemokines and T Helper 1/ T Helper 2 Balance in Humans

**DOI:** 10.1371/journal.pone.0082291

**Published:** 2013-12-11

**Authors:** John Axelsson, Javaid-ur Rehman, Torbjorn Akerstedt, Rolf Ekman, Gregory E. Miller, Caroline Olgart Höglund, Mats Lekander

**Affiliations:** 1 Department of Clinical Neuroscience, Karolinska Institutet, Stockholm, Sweden; 2 Osher Center for Integrative Medicine, Karolinska Institutet, Stockholm, Sweden; 3 Stress Research Institute, Stockholm University, Stockholm, Sweden; 4 Institute of Neuroscience and Physiology, University of Gothenburg, Gothenburg, Sweden; 5 Department of Psychology, University of British Columbia, Vancouver, Canada; 6 Respiratory Medicine Unit, Department of Medicine Solna and Center for Molecular Medicine, Karolinska Institutet and Karolinska University Hospital, Solna, Stockholm, Sweden; 7 Department of Physiology and Pharmacology, Karolinska Institutet, Stockholm, Sweden; 8 Centre for Allergy Research, Karolinska Institutet, Stockholm, Sweden; Hôpital du Sacré-Coeur de Montréal, Canada

## Abstract

**Background:**

Recent studies suggest that acute sleep deprivation disrupts cellular immune responses by shifting T helper (Th) cell activity towards a Th2 cytokine profile. Since little is known about more long-term effects, we investigated how five days of sleep restriction would affect pro-inflammatory, chemotactic, Th1- and Th2 cytokine secretion.

**Methods:**

Nine healthy males participated in an experimental sleep protocol with two baseline sleep-wake cycles (sleep 23.00 – 07.00 h) followed by 5 days with restricted sleep (03.00 – 07.00 h). On the second baseline day and on the fifth day with restricted sleep, samples were drawn every third hour for determination of cytokines/chemokines (tumor necrosis factor alpha (TNF-α), interleukin (IL) -1β, IL-2, IL-4 and monocyte chemoattractant protein-1 (MCP-1)) after *in* *vitro* stimulation of whole blood samples with the mitogen phytohemagglutinin (PHA). Also leukocyte numbers, mononuclear cells and cortisol were analysed.

**Results:**

5-days of sleep restriction affected PHA-induced immune responses in several ways. There was a general decrease of IL-2 production (p<.05). A shift in Th1/Th2 cytokine balance was also evident, as determined by a decrease in IL2/IL4 ratio. No other main effects of restricted sleep were shown. Two significant interactions showed that restricted sleep resulted in increased TNF-α and MCP-1 in the late evening and early night hours (p’s<.05). In addition, all variables varied across the 24 h day.

**Conclusions:**

5-days of sleep restriction is characterized by a shift towards Th2 activity (i.e. lower 1L-2/IL-4 ratio) which is similar to the effects of acute sleep deprivation and psychological stress. This may have implications for people suffering from conditions characterized by excessive Th2 activity like in allergic disease, such as asthma, for whom restricted sleep could have negative consequences.

## Introduction

It is commonly believed that sleep supports immune function, and that lack of sleep increases the risk for infections [1-3]. In modern society, an increasing proportion of the population sleeps less than 5 or 6 hours [4], a trend which seems particularly common in the working population [5]. Despite its societal relevance, there is little understanding of how cumulative sleep restriction affects immune function.

There is strong support that lack of sleep disrupts cellular immunity, as seen in studies of acute total sleep deprivation in healthy humans when typically deprived of sleep for one to three days. Many studies indicate that acute sleep deprivation increases natural killer (NK) cell numbers during the night, but that there is a decrease of both numbers and activity the following day [6-12]. In contrast, if sleep deprivation persists for 60 hours, both NK cell numbers and NK cell activity are increased [7]. This suggests that the effects of sleep deprivation on NK function is related to the degree of sleep deprivation. In addition, the type of sleep deprivation is important for its effects. Studies of phytohaemagglutinin (PHA)-stimulated lymphocyte activity show suppressed reactivity [6,13] or no effects on T cell function [7,9] in response to total sleep deprivation. Naturally occurring short sleep has, on the other hand, been shown to relate to increased T-cell function [12]. These studies are, however, limited to the acute effects of either restricted or total sleep loss. 

Relatively few studies have investigated the effects of sustained sleep restriction. These studies indicate a mild inflammatory upregulation (e.g. IL-6) [14,15] in response to restricted sleep over time, which partly contradicts findings from studies on acute sleep deprivation [16] and habitual short sleepers [17]. Despite some support for a suppressive effects on anti-body production [18] and an increase of PHA-stimulated interleukin (IL)-17 levels [19], there is a clear lack of knowledge of how longer periods of insufficient sleep affects immune function. Thus, there is no systematic knowledge of how sustained periods with sleep restriction affects helper T (Th) cell activity. In addition, there is sparse knowledge about how other immune regulatory markers, such as chemokines, are affected by restricted sleep for longer periods.

The cytokine profiles of Th lymphocytes are classically classified into two functional subgroups, denoted Th1 and Th2 [20-22]. A few studies have found that acute sleep deprivation entails a shift towards Th2 (release of cytokines such as IL-4, IL-5) rather than a Th1 pattern (release of e.g. IFN-γ, IL-2) [22,23]. Although clinical findings suggest that disturbed sleep is associated with a Th2 pattern, as seen in in alcoholics (measured with the IL-6/IL-10 ratio) [24] and insomnia patients (interferon (IFN)-γ/IL-4) [25], there is a lack of experimental studies on the effects of sustained sleep restriction on Th1- and Th2-related cytokines balance and on inflammatory/chemotactic cytokines. 

The aim of the present study was to investigate how 5 days with restricted sleep, resembling a work week with short sleep, affects the production of pro-inflammatory and chemotactic (such as MCP-1) cytokines, as well as cytokines associated with Th1 and Th2 activity, among healthy subjects. Moreover, the present study includes a more thorough blood sampling procedure than many previous studies, with the intention to analyse effects across the entire 24h window.

## Materials and Methods

### Ethics statement

The study was approved by the Regional Ethical Review Board in Stockholm (Dnr 00-238). Written informed consent was obtained from all participants. 

### Study subjects

Nine healthy males (age range 23-28 yrs) with no physical or mental diseases participated in the study. The inclusion criteria were: being a non-smoking male between 18-40 years of age that had a moderate coffee- and alcohol consumption, and having a habitual sleep need between 7.0 and 8.5 hours. The exclusion criteria were any sleep disorders, psychopathology, somatic/affect disorders, all serious somatic disorders, medications or treatments assumed to affect sleep, severe autoimmune disorders or being obese. Any subjects were also excluded if they had worked nights the last 3 months or travelled across more than 1 time zone the last 4 weeks. Recruitment was based on flyers and posters at 2 major universities in the Stockholm area. Thus, all were non-smokers, non-obese, moderate alcohol and coffee consumers, had a normal sleep need (habitual sleep need ranged between 7.0 and 8.5 hours) and were not under medication. Subjects were compensated economically for participation.

### Sleep protocol

**Figure 1:**
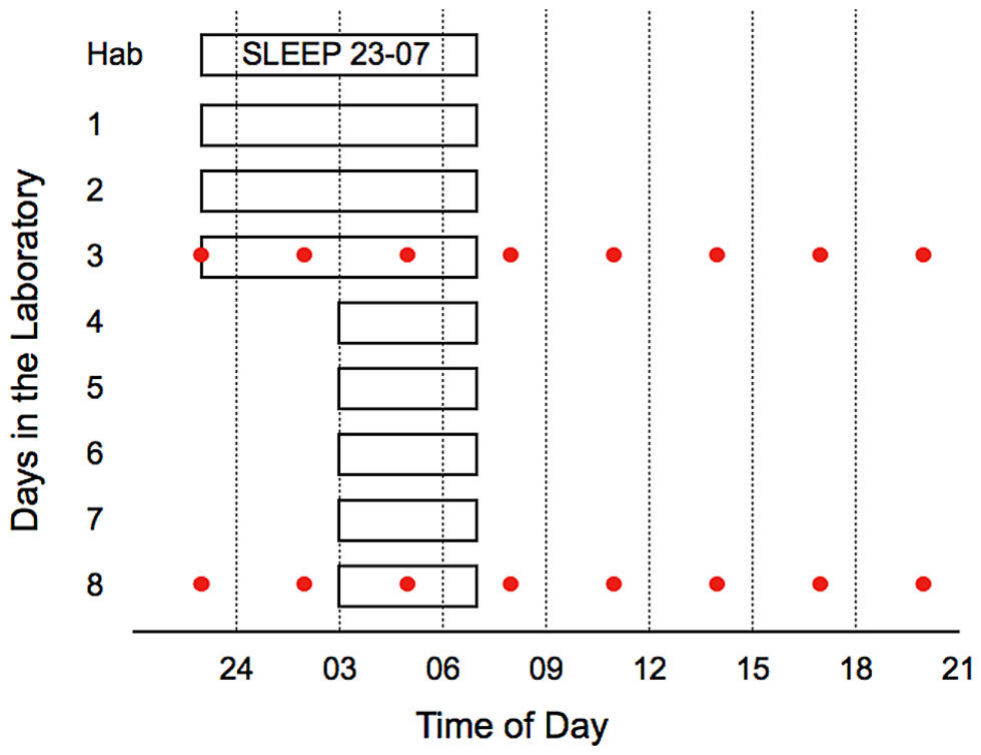
Protocol for participants. Sleep times (white bars with black lines) and blood samplings (circles) during baseline (day 3) and after five days with restricted sleep (day 8). The habituation night (Hab) occurred 4 days prior the data collection period.

Subjects adhered to a sleep schedule with bedtime at 23.00h ± 30min and time of rising at 07.00h ± 30min in their own homes, starting two weeks prior to the first laboratory day (Figure 1). The subjects spent one habituation day (sleep 23.00–07.00 h) in the sleep laboratory at the National Institute of Psychosocial Medicine to become used to the environment and the measures. This was followed by another 4 study/work days with sleep at home (sleep 23.00–07.00 h). Subsequently, subjects spent 10 days and nights in the sleep laboratory with two baseline days (sleep 23.00-07.00 h), five days with sleep restriction (sleep 03.00–07.00 h) and three recovery days (sleep 23.00-07.00 h). To ensure compliance with the sleep protocol, subjects wore actiwatches (Cambridge Neurotechnology, Cambridge, UK) when sleeping at home. In the sleep laboratory, all sleep periods were polysomnographically recorded, as previously reported [26]. In the laboratory, subjects had separate sound-insulated bedrooms and could watch video, play games, read books/magazines, use the internet and were allowed light work or studies. Subjects were outdoors at least twice each day (between 08.30 and 21.00 h) to parallel normal behavior. All meals were consumed during the hour subsequent to each daytime blood sample. The subjects were not permitted smoking or alcohol consumption during the entire protocol and refrained from hard physical activity at least two days before coming to the laboratory.

### Blood sampling procedures

An i.v. catheter was inserted 2 h prior to blood sampling in the evening each day. Venous blood was drawn hourly 23.00-08.00 h, and every 3^rd^ hour 08.00-23.00 h. To avoid interruptions of sleep, samples taken 23.00-08.00 h were drawn from an adjacent room through an i.v. catheter that was kept patent with a slow infusion of NaCl (0.9%). Subjects had been in a supine position for at least 30 minutes prior to all sampling. 

### Analysis of white blood cells

EDTA-samples were collected for complete blood counts with leukocyte differentials. Samples were handed to the Karolinska University Laboratory, Solna, Sweden within 4 hours of sampling and analyzed promptly. White blood cell (WBC) count and differential WBC count (which includes lymphocytes and monocytes) were analyzed by flow-cytometry (ADVIA 120/2120, Siemens).

### Cortisol analysis

Serum samples were placed in room temperature for 30 minutes before centrifugation at 2,600 x *g* for 7 min at 4 °C. After centrifugation samples were pipetted and frozen at – 70 °C until analyzed. Serum cortisol levels were determined by specific immunofluorometric assay (Autodelphia, Wallac Oy, Turku, Finland) with an intra- and inter assay CV of 3.6 % and 1.9 %, respectively.

### Preparation and stimulation of whole blood

Peripheral blood was obtained in heparinized vacutainers every third hour for 24 hours starting at 23.00 h. Whole blood was diluted 1:10 in RPMI 1640, supplemented with 100 U/ml penicillin, 100 μg/mL streptomycin (penicillin-streptomycin), 2 mM L-glutamine (all Sigma-Aldrich, St Louis, MO, USA), and 2 ml blood suspensions were than added to each well in 24-well flat bottom plates (BD Falcon^TM^, Franklin Lakes, NJ, USA). Samples were cultured for 48 h at 37 °C with 5 % CO_2_ in the presence of 10 μg/ml phytohemagglutinin (PHA) (L9132) (Sigma-Aldrich) as previously described (de Groote et al., 1992). Cell culture supernatants were then collected, centrifuged at 400 x *g* for 10 min at 4°C, and stored in aliquots at -70°C until analyzed.

### Analysis of cytokines and chemokines

Cell culture supernatants were analysed for tumor necrosis factor alpha (TNF-α), interleukin (IL) 1β, IL-2, IL-4 and monocyte chemoattractant protein-1 (MCP-1) with automated biochip immunoassay system – Evidence, Randox Laboratories Ltd (Crumlin, UK) [27]. No other cytokines were analysed. For the analyses of TNF-α and MCP-1, samples were diluted 20 times to better fit to the specified detection range of these cytokines with this biochip system. The assays were performed on coded samples by investigators who were blinded to experimental category. 

### Statistical analysis

A mixed effects regression analysis (ANOVA with random effects for individual intercepts) were estimated with STATA 11.1 with the Huyhn-Feldt epsilon correction applied to adjust for violations against the assumption of sphericity. “Condition” (the 2^nd^ baseline day vs. the 5^th^ day with sleep restriction) and “Time of Day” were within-subject variables. Exploratory t-tests were calculated between corresponding time points if there was a significant main effect or interaction. The purpose of these post-hoc t-tests was, together with the figures, to indicate where the main and interaction effects could be found in the 24-hour window. Skewed variables were (log) transformed before analysis. However, all figures are based on raw data. An alpha level of 0.05 was used to test for significance.

## Results

### Cortisol

**Figure 2:**
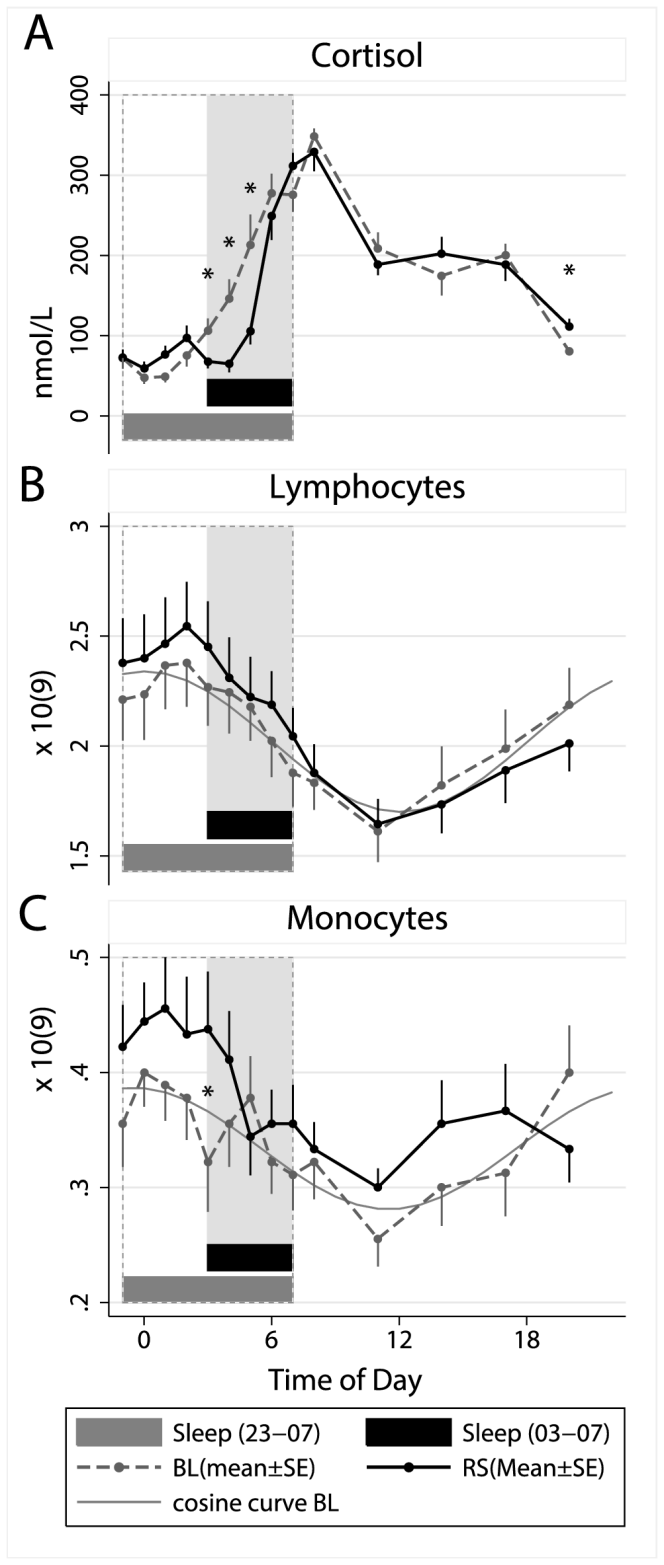
Effects of restricted sleep on cortisol and leukocyte numbers. Means ± SE numbers of (A) cortisol, (B) lymphocytes and (C) monocytes during a baseline sleep-wake cycle (BL=sleep 23.00–07.00 h) and after five days with restricted sleep (RS=sleep 03.00–07.00 h). Grey lines illustrate adapted cosine curves of confirmed circadian rhythm during baseline; shaded area depicts sleep time during restricted sleep (03.00–07.00 h) and dashed box depicts baseline sleep (23.00–07.00 h).

Cortisol showed the expected pattern with significant effects for time of day (p<.0001), with lowest levels at midnight, increasing levels during the latter part of sleep and highest levels at 08.00 h during both conditions (Figure 2A and Table 1). Overall levels did not differ significantly between conditions, but there was a significant interaction between condition and time of day (p<.05). The figure and the exploratory t-tests indicated that this difference was not related to an altered rhythm, but rather to significantly suppressed levels during the first hours of restricted sleep (03.00-05.00 h), as indicated by t-tests (p<.05), when sleep time was delayed to 03.00 h. The comparison of the evening cortisol samples also indicated a slight increase during the restricted sleep condition at 20.00 h (p<.05).

**Table 1:** Effects of restricted sleep on immune cells and stimulated levels of cytokines and chemokines.

Parameters	p-values			
*Hormones*	*Condition*		*Timeofday*	*Interaction*
Cortisol	0.5752		0.0000	0.0442
*Whitebloodcells*				
Lymphocytes	0.1185		0.0000	0.3556
Monocytes	0.0641		0.0001	0.0399
*Cytokines&chemokines*				
TNF-α		0.6613	0.0000	0.0006
IL-1β		0.3300	0.0000	0.1851
MCP-1		0.4038	0.0022	0.0409
IL-2		0.0414	0.0000	0.0692
IL-4		0.1583	0.0000	0.1919
IL-2/IL-4 (ratio)		0.0063	0.0021	0.0322

P-values for ANOVAs comparing Baseline (sleep 23.00-07.00 h) with the 5th day of sleep restriction (sleep 03.00-07.00 h), with respect to circulating levels of cortisol and number of white blood cells, and cytokines and chemokines released from *in vitro*- stimulated white blood cells.

TNF-α=tumor necrosis factor alpha, IL=interleukin, MCP-1=monocyte chemoattractant protein 1. ANOVA=analysis of variance. All p-values have been corrected with Huynh-Feldt Epsilon.

### Lymphocytes and Monocytes

As illustrated in Table 1 and Figure 2B-C, the overall levels of circulating lymphocytes and monocytes did not differ significantly between conditions. A significant interaction showed that monocyte numbers were higher during the first part of night during sleep restriction condition (when subjects were awake), and a t-test confirmed that monocytes were higher for the restricted sleep condition at 03.00 h. Both variables showed strong circadian rhythms with the average acrophase for lymphocytes at 02.00 h under both conditions and for monocytes at 24.00 h during baseline and at 01.00 h during restricted sleep condition (a cosinor curve was fit to the data to illustrate the circadian pattern during normal 8 h sleep). 

### Levels of cytokines and chemokines after *in vitro* stimulation with *phytohemagglutinin (PHA*)

**Figure 3:**
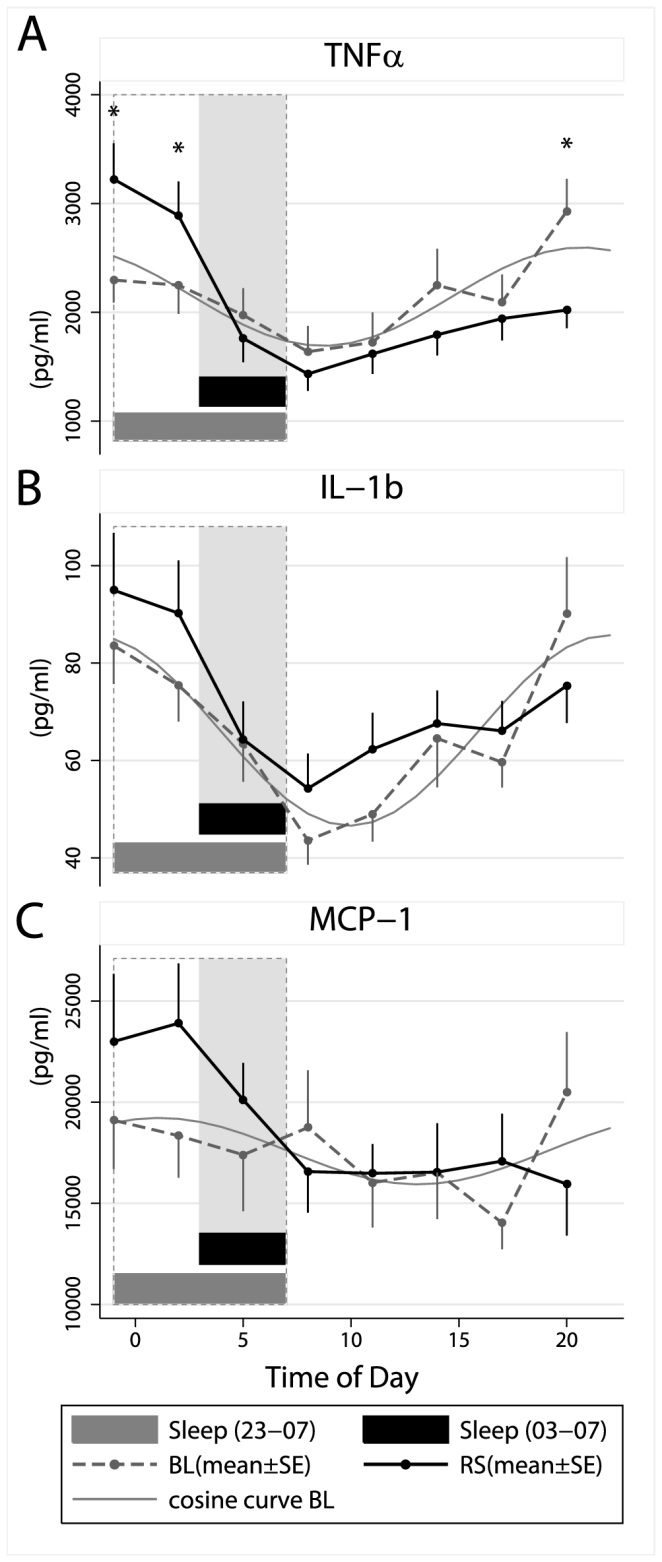
Effects of restricted sleep on stimulated levels of cytokines and chemokines. Means ± SE numbers of (A) tumor necrosis factor alpha (TNF-α), (B) interleukin (IL) 1β and (C) monocyte chemoattractant protein 1 (MCP-1) during a baseline sleep-wake cycle (BL=sleep 23.00–07.00 h) and after five days with restricted sleep (RS=sleep 03.00–07.00 h). Grey lines illustrate adapted cosine curves of confirmed circadian rhythm during baseline; shaded area depicts sleep time during restricted sleep (03.00–07.00 h) and dashed box depicts baseline sleep (23.00–07.00 h).

**Figure 4:**
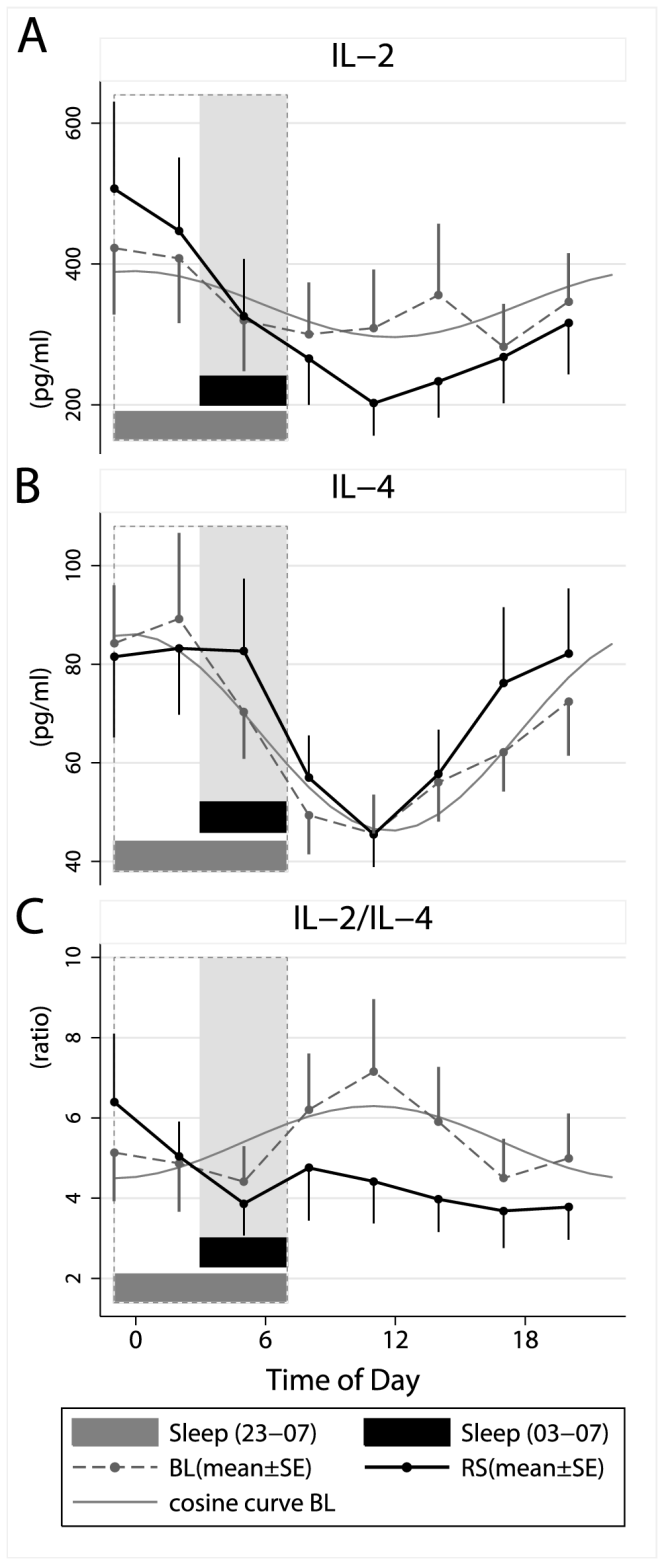
Effects of restricted sleep on stimulated levels of cytokines and T helper 1/T helper 2 balance. Means ± SE numbers of (A) interleukin (IL) 2, (B) IL-4 and (C) IL-2/IL-4 ratio during a baseline sleep-wake cycle (BL=sleep 23.00–07.00 h) and after five days with restricted sleep (RS=sleep 03.00–07.00 h). Grey lines illustrate adapted cosine curves of confirmed circadian rhythm during baseline; shaded area depicts sleep time during restricted sleep (03.00–07.00 h) and dashed box depicts baseline sleep (23.00–07.00 h).

All parameters showed a strong diurnal rhythmicity, which seemed stable during both conditions, perhaps with the exception of MCP-1 (Figure 3 and 4). A significant main effect of condition (p<.05) was observed only for IL-2 (Table 1), levels being lower during restricted sleep (Figure 4A). The interaction between time and condition were significant for TNF-α and MCP-1 (Table 1), levels being increased when the participants were kept awake (i.e. blood samples taken at 23.00 and .02.00 h) during the restricted sleep condition (Figure 3A and 3C), as indicated by the figures and the exploratory t-tests. Notably, the figures indicate similar increases of both TNF and MCP-1 in the late evening and early night, although this was only supported by significant post hoc t-tests for TNF-a.

The IL-2/IL-4 ratio, which gives an indication of the balance between Th1 and Th2 immune balance – was significantly reduced during the restricted sleep condition, particularly during the time awake (Table 1, Figure 4C). The circadian pattern of the IL-2/IL-4 ratio seen during the baseline day was less obvious during restricted sleep.

## Discussion

Five days of sleep restriction affected PHA induced levels of inflammatory mediators. Levels of TNF-α and MCP-1 were elevated across the midnight hours when subjects were kept awake during the fifth day of shortened sleep. The study also demonstrated reduced IL-2 responses, largely confined to daytime, after five days with restricted sleep. This also resulted in a lowered IL-2/IL-4-ratio, suggesting a switch towards Th2 as a result of restricted sleep. The Th2 shift is consonant with findings from acute sleep deprivation [6,22,23] although these studies showed decreased IL-2 responses to PHA during night-time [6], rather than the day-time. The studies have used slightly different indications of Th2-activity (i.e. IL-10 or IL-4), which could be related to the differences in the timing of the effects. Nevertheless, this study confirms that sustained sleep restriction drives a switch towards Th2 as compared to Th1 cytokines, which is similar to the effect of acute sleep deprivation (24) as well as the naturalistic stress of an academic exam [28]. The elevated levels of TNF-α and MCP-1 in the present study may be relevant for cardiovascular disease. There is an increasing awareness of a link between insufficient sleep and cardiovascular disease [29] and it is well known that inflammations are involved in the pathophysiological development of atherosclerosis [30].

There were strong diurnal components for all measured variables. Both TNF-α and IL-1b responses decreased during the night and had their nadirs at 08.00 h during both normal and restricted sleep. The nadir also coincided with the peak of cortisol. The finding that TNF-α responses were particularly high at 23.00 h and 02.00 h, but then fell sharply when subjects slept supports the notion that sleep suppresses TNF-α responses, which is in line with earlier findings [31]. The MCP-1 response was similar to that of TNF-α, although the circadian pattern was less pronounced. As MCP-1 is an inflammatory chemokine playing an important role in regulation of mononuclear cell trafficking from the blood stream into surrounding tissues, the evening increase during restricted sleep suggests an increased inflammatory response during sleep restriction. The mechanism behind these effects evening effects are unknown, but may relate to cortisol. Cortisol was slightly higher in the evening after restricted sleep, which is in line with earlier findings showing that sleep disturbances are related to increased cortisol the subsequent evening [32]. In general, immune responses show strong diurnal rhythms [33], and the mechanisms seem to include sleep dependent aspects (via prolactin), the circadian system (via cortisol) as well as the numbers of natural regulatory T cells and the internal circadian clocks of macrophages [34,35].

An important finding is that the effects are not present across the entire 24-hour cycle, but instead confined to shorter time periods across the 24-hour window. Hence, most of the time there were no obvious effects of restricted sleep reported. These time contingent effects support the use of a frequent blood sampling procedure in order to find representative effects of putative biological significance for health conditions like asthma or cardiovascular diseases, which are more severe in the early morning hours [36,37]. Notably, there were few differences between conditions during the early morning hours (blood samples at 05.00 h), when one would normally expect impact from cortisol and sleep-dependent hormones [38]. An important note is that the study had a relatively good power to detect main effects and interactions, while the t-tests were carried out in an exploratory manner to indicate where the significant main effects or interactions could be found. The t-tests had a relatively lower power so one should be careful with the interpretations from this study alone with regards to where during the day the effects of sleep restriction are manifest.

The inhibitory effects on IL-2 production by five days of restricted sleep were smaller than those of acute total sleep deprivation [6]. This suggests that a total absence of sleep would suppress the production of IL-2 more vigorously or, alternatively, that compensatory mechanisms are activated to regain homeostasis when sleep is restricted over longer periods. A possible compensatory mechanism may be that T-lymphocytes can, by themselves, produce stimulatory factors in an autocrine or paracrine manner [39]. Other compensatory mechanisms - with the purpose to protect the functioning of immune system during sleep loss - could involve the release of immunomodulating and sleep-dependent hormones such as GH and prolactin [40,41] or up/down regulation of genes [42]. 

*In vitro* responses are obviously affected by both number and activity of monocytes/lymphocytes in the blood samples [6]. It is, hence, possible that the increased levels of TNF-α in the evening during restricted sleep were due to increased numbers of leukocytes expressing this molecule. Concurrently, mean numbers of monocytes and lymphocytes were slightly increased during restricted sleep, although not significant. However, the effects on levels of e.g. TNF- α were not changed when compensating statistically for number of monocytes.

The shift towards Th2-activity, as indicated by the decreased IL-2/IL-4 ratio, could be viewed as a stress-related shift [28,43] or a disturbance of the sleep-induced Th1 stimulation. Particularly early nocturnal sleep is associated with augmented levels of GH and prolactin and suppressed levels of cortisol, aspects that collectively support Th1 activity [38]. Hence, the supressed Th2 activity may be related to a reduction of sleep related hormones rather than less sleep or stress, *per se*. The cortisol rhythm seemed to be unaltered during restricted sleep, the acrophases occurring at 08.00 h in both conditions. To establish a robust rhythm, the participants spent time outdoors twice each day (once in the morning and once in the afternoon) and were exposed to low light levels (below 40 LUX) only after 22.00 h during all days. However, it is still possible that a slight delay of the rhythms (i.e. smaller than 1 hour) might have occurred, alhtough undetected since blood drawing was limited to hourly sampling. The main effects seen on cortisol were most likely related to the different timing of sleep. Going to bed at 03.00 h instead of 23.00 h resulted in suppressed cortisol levels at 03.00-05.00 h rather than 23.00-01.00 h. This sleep suppressive effects on cortisol the first hours of sleep are consonant with earlier findings [44]. In addition, restricted sleep resulted in slightly elevated levels in the evening (20.00 h). These cortisol responses mimic a study with similar sleep restriction [32], but contrast, on the other hand, with a study with more modest sleep restriction [14], with only six hours of sleep per night. Moreover, the slight increase of cortisol at 20.00 h, during the restricted sleep period, was accompanied by reduced levels of TNF-α. While it is difficult to exclude a more subtle cortisol-driven Th2-shift, a more plausible explanation would be lower levels of sleep-induced hormones, such as prolactin and GH.

The present study aimed to use an ecologically valid model, keeping good control over the participants’ sleep over an extended period and combine it with a frequent blood sampling procedure uncovering the circadian rhythmicity of included parameters. The main limitations are the low number of participants, and that only healthy male adults participated. The homogenous group was necessary and the few participants were thoroughly studied in both conditions (8 samples per person in each condition respectively were included). As such, the participants represented their own controls in a repeated measures design, which minimises the interindividual differences. As baseline in all cases was measured before sleep restriction it is possible that order or laboratory effects may have affected our results. To reduce such influence, the participants were acclimatized to the laboratory environment before the start of the baseline measurements, and were also outdoors twice each day (in the morning and the afternoon) to avoid a drift of the circadian system. In addition, the present study design has previously been validated in several previous studies [14,45]. 

The clinical relevance of altered cytokine levels may concern both pathophysiological conditions characterized by disturbed sleep and conditions that involve an altered balance between Th1 and Th2 cytokines. In healthy individuals it has, for example, been shown that self-reported short or disturbed sleep is related to increased risk for virus infections [2]. The results also have implications for individuals with clinical conditions, particularly for individuals suffering from condition with excessive Th2 activity such as asthma [46]. Besides the predominant Th2 activity, inflammatory conditions like asthma are also characterized by the upregulation of chemokines like MCP-1 [47], a trend which is quite evident in our study during the early morning hours when subjects were not allowed to sleep. MCP-1 recruits circulating Th2 cells to the airways and enhances Th2 cell differentiation [48]. It is thus possible that an upregulation of MCP-1 during restricted sleep may increase risk for asthma related exacerbations and that patients with a propensity for Th2-related conditions like asthma should be careful with insufficient sleep. Moreover, the present data is consonant with the proposition that sleep interventions should be considered in chronic inflammatory conditions characterized with Th2 overactivity [49]. Besides the growing body of evidence showing that sleep deprivation leads to a Th2 overactivity amongst humans, further studies are needed to investigate whether this may relate to findings (largely limited to animals) that sleep deprivation may also cause an increase of anti-inflammatory cytokines [16,50], being immune suppressive [51,52] and leading to an incomplete antioxidant protection [53].

In conclusion, we report that repeated sleep restriction was characterized by higher mitogen-stimulated levels of pro-inflammatory agents as TNF-α and MCP-1, and a shift towards Th2 activity, as reflected by an altered Th1/Th2 cytokine balance. These findings corroborate earlier observations displayed after acute sleep deprivation and stress. Consequently, good sleep hygiene may have positive health effects for individuals suffering from conditions characterized by excessive Th2 activity. 
